# Recent Evidence for Orthobiologics Combined with Hydrogels for Joint Tissue Regeneration: Focus on Osteoarthritis

**DOI:** 10.3390/gels11070551

**Published:** 2025-07-17

**Authors:** Carola Cavallo, Giovanna Desando, Martina D’Alessandro, Brunella Grigolo, Livia Roseti

**Affiliations:** Laboratorio RAMSES, Dipartimento Rizzoli Research & Innovation Technology, IRCCS Istituto Ortopedico Rizzoli, 40136 Bologna, Italy; carola.cavallo@ior.it (C.C.); martina.dalessandro@ior.it (M.D.); brunella.grigolo@ior.it (B.G.); livia.roseti@ior.it (L.R.)

**Keywords:** orthobiologics, hydrogel, osteoarthritis, MSCs, BMC, SVF, exosomes, extracellular vesicles, growth factors, PRP

## Abstract

Osteoarthritis is a significant global problem, causing pain and limitations, and contributing to socioeconomic expenses. The etiopathogenesis of this disease encloses genetic, biological, and mechanical aspects. Regenerative medicine, utilizing tissue engineering, has opened the way to new therapeutic approaches employing various orthobiologics. Combined with hydrogels, these compounds may represent a notable option for treating degenerative and inflammatory lesions in OA. The review reports on the main orthobiologics used in preclinical and clinical studies, as well as their association with various types of natural and synthetic hydrogels. Research may increasingly focus on tailored therapies adjusted to suit the joint involved and the severity of the pathology encountered in each patient.

## 1. Introduction

Osteoarthritis (OA) represents the most common chronic disease affecting the elderly as well as the middle-aged population; it is rare in people under 40 but is common in people over 65 years of age. OA may occur due to traumatic injury of the joint and is correlated with factors such as mechanical influences, the effects of aging on cartilage matrix composition and structure, an excessive body mass index, infectious agents that induce joint destruction, and genetic factors [1,2,3]. The discomfort and other indicators linked with OA can significantly diminish a person’s quality of life. This impact extends to both their physical capabilities and their mental well-being.

OA should not be viewed solely as a cartilage issue; it is a long-term condition impacting the entire joint characterized by synovial inflammation, oxidative stress, apoptosis in chondrocytes, cartilage extracellular matrix (ECM) degradation, subchondral bone sclerosis, and osteophyte formation leading to stiffness of the whole joint, pain, and joint failure [4,5].

Although this pathology is widespread, understanding the biological processes that drive OA remains elusive. In recent years, synovial inflammation has become a focal point in understanding its etiology and pathogenesis. Studies have confirmed that inflammation in the synovium of OA patients is closely associated with disease severity and progression [6].

Conventional OA treatments focusing on managing pain and improving function are based on conservative and surgical procedures; in particular, non-pharmacologic approaches like regular exercise, physical and occupational therapies, weight management, and the usage of assistive devices and lifestyle changes such as healthy diet, quitting smoking, and managing stress; various medications; pharmacological therapies that may include the use of paracetamol, topical or oral non-steroidal anti-inflammatory drugs (NSAIDs), or intra-articular corticosteroids; surgical procedures, specifically joint replacement, that represents an option for severe pain that does not improve with other treatments in the late stages [7].

Recently, new therapeutic strategies which focus on protecting and restoring the tissues’ physiological joint structure and activity have emerged as promising strategies for delaying the progression of musculoskeletal diseases, including OA. These treatments are based on regenerative medicine and tissue engineering [8,9].

Regeneration occurs when damaged tissue is replaced by new growths that restore its original architecture and function [10]. Cartilage regeneration is challenging because this tissue has a low regenerative ability due to its avascular, aneural, and free-of-lymphatic vessel structure. Bones have high regenerative ability, but the challenge remains for large tissue loss and pseudo-arthrosis [11].

Tissue engineering combines scaffolds/biomaterials, cells, and soluble/mechanical factors into structures that, once implanted, can regenerate diseased joint parts. Engineered tissues for implantation can be created through static or dynamic (bioreactors) cell seeding onto the scaffold [12]. Due to their anti-inflammatory and healing properties, hydrogels are among the biomaterials engineered to be implantable or injectable for the regeneration of joint tissues in the treatment of OA. Those with healing and anti-inflammatory characteristics have been associated with hydrogels for regenerative purposes in OA management. Compared to surgery, orthobiologic-laden hydrogels are less invasive, reducing recovery time [13].

This narrative review aims to critically analyze preclinical and clinical studies on hydrogels combined with orthobiologics, with a focus on tissue regeneration applications in OA. A search of the MEDLINE and Web of Science databases from November 2024 to March 2025 was conducted. We run the search using the keywords “orthobiologics”, “hydrogels”, osteoarthritis”, mesenchymal stem cells”, “bone marrow”, adipose tissue”, “exosomes”, “extracellular vescicles”, “Growth factors”, and “Platelet Rich plasma”. Non-English sources, conference abstracts, and non-peer-reviewed sources were excluded.

## 2. Orthobiologics

The field of orthopedics has seen a significant increase in treating musculoskeletal disorders through biological substances, commonly known as orthobiologics [14]. Orthobiologics display a low-invasive nature, significant ability to facilitate healing, and affordability as a non-surgical intervention strategy.

Nowadays, clinicians utilize orthobiologics, including chondrocytes, Mesenchymal Stem Cells (MSCs) from bone marrow, adipose tissue, and other sources (synovial tissue, tooth, peripheral blood), induced pluripotent stem cells (iPSCs), Platelet-Rich Plasma (PRP), Bone Marrow Concentrate/Aspirate (BMC/BMA), stromal vascular fraction (SVF), growth factors (GFs), extracellular molecules, and extracellular vesicles (EVs).

**Figure 1:**
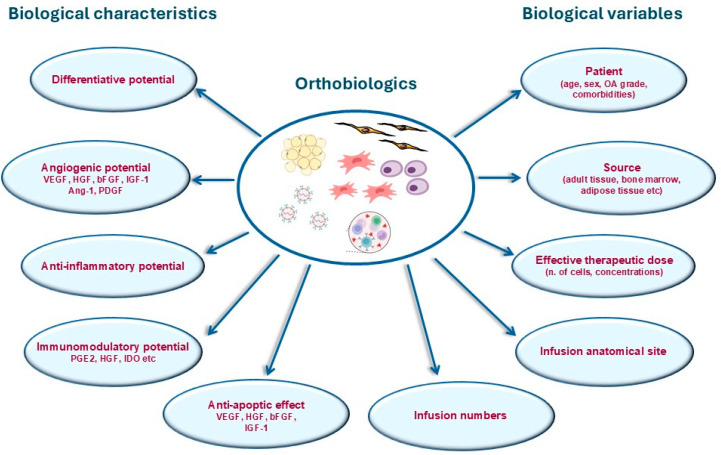
The main biological characteristics and variables of orthobiologics with interest in regenerative medicine.

Although different, these compounds share several important biological functions, with interest in regenerative medicine such as differentiative, angiogenic, anti-inflammatory, immunomodulatory potentials, and anti-apoptotic effects [14]. The variables that can critically affect their efficacy include differences in patient age, sex, or associated medical comorbidities, tissue source, effective therapeutic dose, infusion anatomical site, and number of infusions (Figure 1).

Over the past few decades, scientists have been attempting to identify tissue-specific markers that would aid in more accurately evaluating the efficacy of regenerative processes following orthobiologics treatments (Table 1). Most preclinical and clinical studies showed encouraging results [15].

**Figure 2:**
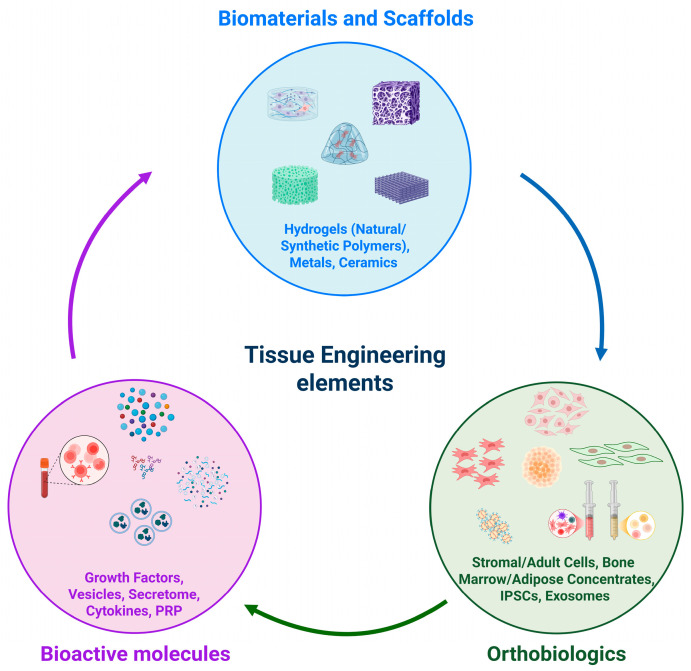
Tissue engineering main protagonists. The image was created with BioRen-der.com (Website homepage: https://www.biorender.com/, accessed on 12 May 2025).

Tissue engineering techniques that merge orthobiologics with biomaterials designed to release substances present a promising avenue for enhancing the treatment of OA (Figure 2).

To establish this ground-breaking therapeutic approach as standard treatment for OA, extensive evaluation of elements, such as diverse biomaterials, cellular components, biological factors, and pharmaceutical agents designed to alter the progression of the disease, is imperative [16].

One of the primary objectives is to directly target and mitigate the inflammatory processes within the affected joints using specialized delivery vehicles designed to facilitate prolonged release of the medication. Within this research field, several investigations focused on formulating self-curing and injectable substances suitable for the intra-articular delivery of regenerative substances.

Orthobiologics should be defined by specific and measurable markers of purity, potency, and biological activity, which aid in their identification, classification, and understanding of their effects on specific tissues and pathologies. However, as highlighted by Rodeo, the variability in the composition and biological activity of different formulations, as well as the batch-to-batch differences, complicates the characterization of orthobiologics, resulting in challenges when interpreting the outcomes of studies [17].

Hydrogels hold potential in OA treatment, not merely because they deliver different orthobiologic products but also because their properties and structures make them ideal candidates for injection.

Combining hydrogels with orthobiologic compounds may enhance their therapeutic efficacy. Moreover, their intra-articular application reduces the risk of side effects due to systemic therapies, such as gastrointestinal bleeding and myocardial infarction [18].

**Table 1:** Principal biomarkers used to evaluate the regenerative efficacy of orthobiologic treatments.

Biomarkers	Type	Clinical Significance	Ref.
CD44, CD151, CD105, CD271	Surface chondrogenic markers	Present on the surface of cells, help to identify cells with chondrogenic potential	[19]
SOX-6, SOX-9	Intracellular chondrogenic markers	Transcription factors that play key roles in cartilage development	[20]
Collagen type II, proteoglycans	Secreted chondrogenic markers	Principal components of hyaline cartilage	[19]
Procollagen type II C-terminal propeptide (PIICP)	Secreted chondrogenic marker	A biomarker of collagen type II synthesis released when cartilage cells produce new collagen	[19,21]
COMP (Cartilage oligomeric matrix protein)	Cartilage metabolism	A marker of cartilage synthesis and degradation, reflecting overall cartilage health	[19]
Alkaline Phosphatase, Collagen Type I, Bone Sialoprotein	Early osteogenic markers	Osteoblast differentiation and activity	[22]
Osterix, Osteocalcin, Osteopontin	Late osteogenic markers	Late marker of bone formation, associated with mineralization and bone remodeling	[22]
Runx-2, TGF-β, BMP (Bone Morphogenetic protein)	Osteogenic markers	Signaling molecules that induce osteogenic differentiation	[19]
VEGF (Vascular endothelial growth factor)	Cell growth, differentiation	Important for promoting angiogenesis and tissue repair	[23]
Procollagen Type I N-terminal Propeptide (P1NP)	Bone formation	A peptide released during collagen synthesis by osteoblasts, indicating bone formation	[22]
hs-CRP (High-sensitivity C-reactive protein)	Inflammation	Elevated levels are associated with inflammation and can predict cartilage loss in OA	[24]
IL-6, TNF-alpha	Inflammation	Cytokines that play a role in inflammation and can be measured in synovial fluid or serum of OA patients	[25]
Chemokines (MCPs, MIP-1α, RANTES, Eotaxins and their receptors)	Inflammation	Molecules involved in the OA pathogenesis	[26]

## 3. Hydrogels

Hydrogels are polymeric materials with a three-dimensional network of hydrophilic macromolecules connected by chemical or physical crosslinks. Their fundamental characteristic is their ability to absorb and retain solvents reversibly, particularly significant amounts of water, ranging from 10 to 20% up to thousands of times their initial dry weight. This “swelling” process involves a marked increase in the volume of the hydrogel [27].

Hydrogels were invented and proposed in the 60s by chemists Otto Wichterle and Drahoslav Lim, who created soft contact lenses made of hydrogel [28]. Since then, these materials have generated tremendous interest in academic and industrial fields. They can be synthesized in different forms, including microparticles, nanoparticles, sheets, and coatings. Their applications range from hygiene products to agriculture and food, as well as the pharmaceutical and biomedical fields. Their characteristics, such as high-water content, softness, flexibility, and porosity, make hydrogels similar to biological tissues, both chemically and mechanically, compared to any other synthetic biomaterials [29]. In the biomedical field, hydrogels are utilized in tissue engineering, regenerative medicine, diagnostics, controlled drug administration, wound and burn treatment, the separation of biomolecules or cells, and as a barrier material to regulate biological adhesion, as well as in biosensors [30]. The swelling process of hydrogels is contingent upon pH, temperature, ionic strength, and electromagnetic radiation; furthermore, the material properties, including shape, flexibility, opacity, and porosity, can be modified by adjusting the parameters listed. When a dehydrated or freeze-dried hydrogel is placed in an aqueous solution, its volume increases due to the swelling effect, which is caused by the retention of water in the solution that remains trapped within the material’s three-dimensional network. As soon as absorption begins, the first water molecules hydrate the groups with greater polarity and hydrophilicity, a quantity defined as primary bound water. The network, therefore, swells and exposes the hydrophobic groups, which interact with the secondary bound water [31].

Primary and secondary bound water combination is referred to as “total bound water.” When the hydrophilic and hydrophobic groups are completely hydrated, the hydrogel absorbs additional water, named free water, which fills the free spaces inside the network [32].

The swelling capacity of hydrogels results from hydrophilic functional groups, like hydroxyl group −OH, carboxylic group −COOH, and amino group −NH_2_, in the polymer (or polymers) used in their formation. Furthermore, depending on factors such as the properties and type of polymer, the molecular weight, and the degree of crosslinking [33], these materials can contain different amounts of water: usually, in the swollen configuration, the mass fraction of water in the hydrogel is much higher than the mass fraction of the polymer [30].

The appropriate parameters for characterizing and evaluating different hydrogels are those that describe the basic structure, the average molecular weight between two adjacent crosslinks, their physical or chemical nature, and pore size. Also, the density of the crosslinks and the polymer’s volume fraction in the swollen state is a property that influences the hydrogel’s behavior [34].

### 3.1. Properties of Hydrogels

Every hydrogel has distinct and unique properties that make it suitable for various applications [35].

#### 3.1.1. Biodegradability

This property makes hydrogels suitable candidates for use as implants in regenerative applications like Poly Lactic Acid and Poly Lactic-co-Glycolic Acid -PLGA- and Poly (vinyl alcohol) -PVA-) and for drug delivery applications [27].

#### 3.1.2. Biocompatibility

Hydrogels possess hydrophilic surfaces, which minimize the interfacial tension with body fluids and adjacent tissues. Poly(lactide-co-glycolide) (PLG) and Poly (lactic-Co-Glycolic Acid) (PLGA) are widely used materials [36].

#### 3.1.3. Controlled Drug Delivery Rate

Hydrogels function as a mini reservoir for drugs, releasing them at a slower rate. Moreover, their pH responsiveness makes them excellent candidates for site-specific drug delivery devices, e.g., poly(ethylene glycol) (PEG) [37,38].

#### 3.1.4. Thermoplasticity and Viscoelasticity

Hydrogels, due to their crosslinks, swell significantly, and this expansion contributes to their viscoelastic and elastic properties. This flexibility and elasticity, along with stickiness, resembles the morphology of natural tissues (for example, Poly (ethylene oxide) (PEO)-based hydrogels [35,39].

#### 3.1.5. Non-Toxic By-Products

Hydrogels such as poly(lactic acid) (PLA), poly(lactide-co-glycolide) (PLG), and poly lactic-co-glycolic acid (PLGA) degrade over time, leaving harmless, non-toxic by-products that are subsequently metabolized and excreted by the body’s metabolic pathways [35].

### 3.2. Classification of Hydrogels

**Figure 3:**
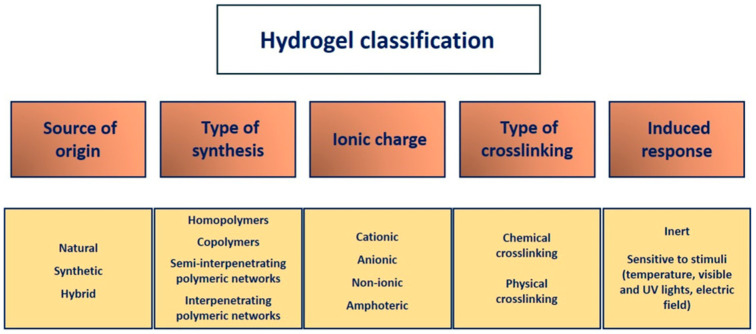
Scheme representing hydrogel classification. Hydrogels are classified according to their source of origin, type of synthesis, ionic charge, type of crosslinking, and induced response.

The classification of hydrogels is based on several criteria: they can be categorized by their source of origin, method of synthesis, type of crosslinking, and ionic charge on bound groups (Figure 3). A more advanced category includes hydrogels that are responsive to pH, temperature, electric fields, light, and substrate [32,33,35].

#### 3.2.1. Source of Origin

Hydrogels can be classified based on the origin of the polymer that constitutes them as natural, synthetic, or hybrid [40].

Natural hydrogels are biocompatible, biodegradable, and non-toxic to the human body, making them suitable for biomedical applications. They include proteins such as collagen and gelatin, polysaccharides like cellulose, and chitosan. The main drawbacks are the low mechanical strength, thermal stability, susceptibility to degradation, and ‘burst effect’ during drug delivery [41].

Synthetic hydrogels exhibit better mechanical properties, resulting in slower degradation compared to natural ones. They also have durability, which increases their potential applications in the industrial field [42]. They also have limitations, which restrict their use in the biomedical field. To overcome these limitations, hybrid hydrogels containing natural and synthetic materials are synthesized with multiple functionalities and advanced chemical, physical, and biological properties.

#### 3.2.2. Type of Synthesis

Depending on their synthesis type, the three-dimensional architecture of hydrogels can be homopolymeric, copolymeric, semi-interpenetrating (semi-IPNs), and interpenetrating polymer networks (IPNs) [35].

Homopolymer hydrogels consist of polymer chains composed of a single type of monomer, and they are already used in the production of contact lenses and wound healing dressings [43]. However, due to their limited functionality, they do not meet the requirements for biomedical and drug delivery applications [44].

Copolymer hydrogels consist of two or more monomers, at least one of which is hydrophilic. They can be physically or chemically crosslinked using various polymerization techniques [44].

Semi-interpenetrating polymeric networks (semi-IPNs), also known as alloys of crosslinked hydrogels, involve blending two hydrogels, with one being crosslinked in the presence of the other, leading to additional interactions between them [22]. They possess high mechanical strength [44].

IPNs consist of intertwined polymer networks, in which the first network swells in the monomer of the second, followed by crosslinking to create a highly compact three-dimensional network structure. These hydrogels are dense matrices with stiffer and more rigid mechanical properties, making them more efficient than conventional hydrogels in drug-loading applications [44].

#### 3.2.3. Type of Crosslinking

The crosslinking process encompasses the polymer solution and transforms it into a gel, restricting the movement of the polymeric chains. As a result, the 3D structure of the hydrogel is maintained, and its mechanical properties are enhanced [35].

The type of crosslinking between the chains [34] determines the classification of physical and chemical hydrogels [19,33].

Physically crosslinked hydrogels are also known as ‘reversible gels,’ as when placed together, weak forces like ionic and hydrogen bonding, or hydrophobic interactions, operate between the molecules. Such secondary bonding forces can be disrupted by applying stress or by changing surrounding conditions [44].

Chemically crosslinked hydrogels, or ‘permanent gels,’ are formed through crosslinking strong covalent bonds in the hydrogel matrix, along with hydrogen bonds, which leads to the creation of a crosslinked network. Graft copolymerization, chemical crosslinking achieved through radical polymerization utilizing redox and ionic polymerization systems, and UV and gamma radiation have been employed [44].

#### 3.2.4. Ionic Charge

Hydrogels can be classified based on the presence or absence of electrical charge as cationic, which have positive charges; anionic, which have negative charges; amphoteric, which have both positive and negative charges; and non-ionic or neutral, which have no charges [35,45].

Ionic hydrogels respond to pH because the ionic chains dissociate to varying degrees at different hydrogen ion concentrations. Cationic hydrogels, which carry positive charges, swell at lower pH values.

Anionic hydrogels with negative charges dissociate maximally at higher pH values and exhibit maximum swelling in a basic medium.

Hydrogels made from vinyl pyridine, aminoethyl methacrylate (AEMA), and diethylaminoethyl methacrylate (DEAEMA) monomers are cationic.

Hydrogels that strongly depend on pH changes are amphoteric due to acidic and basic groups equilibrium at the isoelectric point [44].

#### 3.2.5. Induced Response

Light, pH, glucose, enzymes, and specific ligands can significantly alter hydrogel behavior during the swelling process.

Positive temperature-responsive hydrogels undergo phase changes at or above their upper critical solution temperature, contracting when cooled below this threshold. In contrast, negative temperature-responsive hydrogels have a lower critical solution temperature and shrink when heated beyond this point. Physically crosslinked thermo-responsive hydrogels exhibit sol–gel phase transitions, while chemically crosslinked hydrogels undergo volume changes due to temperature fluctuations [44].

Photo-responsive hydrogels are categorized into two types: UV- and visible-light-responsive. Unlike UV light, visible light is easily accessible, cost-effective, non-toxic, and clean, making it a straightforward and safe option for handling. Therefore, light-responsive hydrogels prove to be more practical than UV-responsive ones. The main functional groups that contribute to photoresponse include azobenzene, triphenylmethane, and spropyran. Conversely, UV light-responsive hydrogels are synthesized by incorporating a leuco-derivative molecule, specifically bis (4-methylamino) phenylmethylleucocyanide, into the hydrogel matrix [46].

Electroresponsive hydrogels demonstrate swelling and deswelling behavior in response to an electric field. Synthetic and natural hydrogels, combined with polyanions, polycations, and amphoteric polyelectrolytes, can be utilized to create electroresponsive hydrogels [44].

## 4. Hydrogels for OA Treatment

Hydrogels have been used in medical research and life science thanks to their controllable mechanical properties, biocompatibility, stability, hydrophilicity, biodegradability, and low toxicity [13]. These characteristics also made them suitable for treating OA [47,48], leveraging the characteristics of the hydrogel material to address the varying manifestations of OA. Thanks to the characteristics of lubrication and flowability, hydrogels permit direct injection into the articular space to ameliorate the friction experienced during joint articulation, thereby mitigating the pain and mobility symptoms associated with the disease.

One of the most used types of hydrogels employed in OA management is Hyaluronic acid (HA). Clinical applications of this substance have led to considerable alleviation of OA symptoms. Moreover, HA has proven advantageous in extending the period before a patient undergoes arthroplasty [49].

The other approach involves using hydrogels as vehicles for biological compounds, such as the orthobiologics as mentioned earlier, to stimulate a regenerative process. It achieves this by incorporating cells or therapeutic agents into the material before administration or by attracting cells to the injury site after the hydrogel has been introduced alone. This necessitates a custom-made approach to the architecture of hydrogel scaffolds.

**Figure 4:**
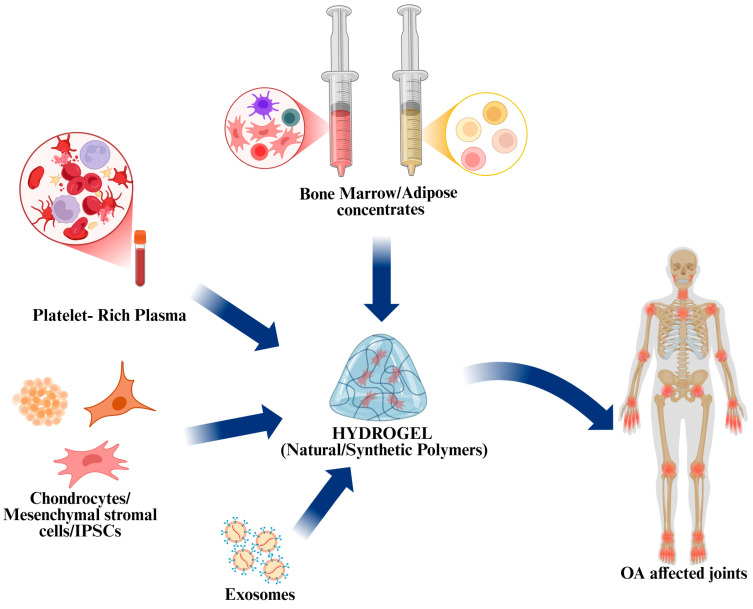
Image of orthobiologics used in clinical practice and/or investigated for OA treatments. These include chondrocytes, Mesenchymal Stem Cells (MSCs) from bone marrow, adipose tissue, and other sources (such as synovial tissue, teeth, peripheral blood), induced pluripotent stem cells (iPSCs), platelet-rich plasma, bone marrow and adipose tissue concentrates, and exosomes. The image was created with BioRender.com (Website homepage: https://www.biorender.com/, accessed on: 12 May 2025).

Hydrogels are among the numerous scaffolds used for regeneration. Hydrogels are biocompatible and biodegradable biomaterials that offer several advantages for treating OA. They can provide structural support to articular tissues, helping to alleviate joint pressure, reduce friction, and improve joint movement. Many hydrogels exhibit anti-inflammatory properties, which help relieve pain, swelling, and stiffness. Moreover, they can favor and enhance regeneration by mimicking the structural and functional features of naive joint tissues, carrying and interacting with cells or other biological compounds (orthobiologics) added ex vivo or present in the inflamed articular microenvironment. Finally, hydrogels can deliver drugs and bioactive substances directly to the affected joint, ensuring sustained release and targeted treatment [47] (Figure 4).

### 4.1. Expanded Cells Combined with Hydrogels for OA Treatment

As already underlined, hydrogels are promising scaffolds in joint tissue engineering applications: their high water content and permeability make them suitable for cell loading [50]. The cellular part is also crucial since it helps promote tissue formation by differentiating or releasing specific soluble molecules [51].

The interaction between chondrocytes and hydrogels can enhance the regeneration process. For example, a chitosan hydrogel-demineralized bone matrix mixed scaffold promoted the upregulation of allogeneic chondrocyte regeneration genes in a rabbit model [52]. In another example, α-cyclodextrin and polyethylene glycol (PEG) incorporated into a gelatin covalent matrix and implanted with chondrocytes influenced the thermo-mechanobiology of cartilage, which significantly contributed to the lesion regeneration [53].

A hydrogel based on a combination of methacryloyloxyethyl-phosphorylcholine and a novel sulfobetaine-methacrylate-based monomer (zwitterionic monomer solution) was able to infiltrate into human OA cartilage explants to replace lost matrix proteoglycans, forming zwitterionic cartilage–hydrogel areas [54].

MSCs are investigated and utilized in clinics due to their regenerative properties, such as anti-inflammatory, anti-apoptotic, and immunoregulatory effects, which make them promising candidates for treating OA [55]. In contrast, embryonic stem cells and iPSCs may raise ethical concerns [56].

Hydrogels can enhance the musculoskeletal regenerative ability of MSCs by prolonging their survival and retention in the injured site. Examples of natural hydrogels investigated are polysaccharides like HA, agarose, alginate, glycosaminoglycans, and chitosan, proteins like collagen, elastin, and gelatin, and synthetic hydrogels formed from polymers, such as polyvinyl alcohol (PVA), Poly(lactide-co-glycolide) (PLGA), or a combination of them [57,58,59].

### 4.2. Concentrated Cells Combined with Hydrogels for OA Treatment

Even with positive outcomes in clinical settings, employing isolated chondrocytes or MSCs presents a key hurdle, including the necessity of two separate surgical interventions and the considerable financial burden involved. BMC and SVF are orthobiologic therapies considered alternatives to expanded cells for regenerative therapies.

The inherent properties of pluripotent cells are not only dictated by the cells’ internal characteristics but also shaped by their relationships with their surroundings, defined as a physiological niche which offers signaling systems, encompassing elements such as the extracellular matrix (ECM), adhesion molecules, growth factors, cytokines, and chemokines [60]. These factors, in turn, are released by the cells already present within the niche.

BMC and SVF’s potential, encompassing not only regenerative capabilities but also anti-inflammatory properties, makes them a potentially appealing avenue for cartilage regeneration when dealing with OA [59].

Hydrogels can enhance the regenerative properties of these biological compounds. For example, a construct made of autologous SVF loaded onto an HA/gelatin-biphasic calcium phosphate (HA-Gel/BCP) scaffold showed higher bone regeneration ability than the unloaded HA-Gel/BCP scaffold when implanted in a rat skull critical-size defect model. The contribution of the hydrogel to regeneration was significant. BCP exhibits osteoconductive properties; however, its mechanical properties are compromised due to its high porosity. As a typical polymeric complex, HA/gelatin hydrogel has unique architectural features and biological properties that could enhance cellular attachment, proliferation, and migration, avoiding structure collapse in vivo [61].

### 4.3. MSCs-Derived Molecules Combined with Hydrogels for OA Treatment

MSCs are multipotent cells present in adult tissues [62]. They can differentiate into mesodermal, ectodermal, and endodermal lineages. MSCs serve as supportive stromal cells in the body, maintaining tissue homeostasis by detecting environmental changes and restoring equilibrium. In pathological conditions such as OA, MSCs respond to signals from the damaged areas, where they can migrate [55].

The evidence of MSCs’ differentiation potential in vitro has led to the hypothesis that MSCs could contribute to tissue regeneration through differentiation when transplanted into a host. However, this perspective has been debated in recent decades. Many studies have demonstrated that MSCs cannot be grafted in adequate numbers and for a long enough period to allow tissue regeneration by differentiation, as observed in vitro [63].

Instead, a new hypothesis gained ground: the beneficial effect of transplanted MSCs was related to their secretory (paracrine) activity rather than their differentiation potential. Arnold I. Caplan suggested that the acronym MSCs should stand for “Medicinal Signaling Cells,” which better represents their active role in signaling [64].

MSCs exert their trophic, anti-inflammatory, anti-fibrotic, anti-apoptotic, and neoangiogenic effects by producing and secreting into the extracellular space a rich and complex set of biologic components known as the “secretome” [65].

The secretome offers similar benefits to MSC-based therapies, but with fewer risks, including immune compatibility and tumorigenicity. Additionally, secretomes are more scalable and have a longer shelf life. The secretome can be obtained from the culture medium of MSCs and can be genetically, biochemically, or physically modified to enhance its therapeutic efficacy for specific applications [66].

The secretome includes two main components: soluble and vesicular. The soluble component encloses bioactive molecules and biological factors (GFs, cytokines, chemokines, lipids, and genetic material). The insoluble part encloses EVs such as exosomes, microvesicles (MVs), and apoptotic bodies. MVs are produced by healthy cells, and apoptotic bodies are made by dying cells and are not considered in this review [67].

### 4.4. Exosomes Combined with Hydrogels for OA Treatment

Exosomes are small EVs ranging in diameter from about 40 to 160 nanometers, with an average size of about 100 nanometers. EVs protect their contents, such as mRNAs, miRNAs, and proteins, from degradation and deliver them to target recipient cells [68].

Exosomes are produced through the endosomal pathway: after the inward budding of the cell membrane, early endosomes develop into multivesicular bodies (MVBs) via the inward movement of their membrane and the formation of intraluminal vesicles [67]. Exosomes can either be degraded by lysosomes or released to the ECM through fusion with the cell membrane. In most cells, the endocytosome sorting and transport complex (ESCRT) [69] is primarily involved in the production and release of exosomes. MVs, on the other hand, come from the outward budding of the plasma membrane by the action of the cytoskeleton [68].

Exosomes contain a variety of molecules, including cytokines, proteins, lipids, and non-coding RNAs. These molecules enable exosomes to participate in multiple physiological processes by facilitating cell-to-cell communication and several pathological processes, where they play a role in the immune response, inflammation, cell senescence, apoptosis, and differentiation [70].

Exosomes have several advantages over both traditional and cell-based therapies. Their low immunogenicity reduces the risk of immune rejection, a common concern with stem cell therapies [29]. Exosomes are also easier to store, transport, and administer than living cells. They are acellular and, therefore, pose no risk of tumorigenicity, making them safer for long-term use [40].

Exosomes can derive from different types of cells, including MSCs from adipose tissue and bone marrow, T and B lymphocytes, dendritic cells, mast cells, platelets, and cancer cells. They are not restricted to specific body compartments. They can be found in various fluids such as blood, urine, cerebrospinal fluid, milk, and saliva, making them accessible for research and potential therapeutic applications [68,70].

Chondrocytes, synovial fibroblasts, osteoblasts, MSCs, and tendon cells can produce and secrete microRNAs (miRNAs), long non-coding RNAs (lncRNAs), and circular RNAs (circRNAs). These are the three major types of ncRNAs that interact with each other and with miRNAs, regulate mRNA expression, and, thus, the physiological state of cells [71]. Exosomes derived from bone marrow MSCs were demonstrated to promote osteogenesis [72] and angiogenesis through the activation of the BMP-2/Smad1/RUNX2 and the HIF-1α/VEGF pathways in femoral nonunion fractures in a rat model [73].

During OA, exosomes undergo changes in association with disease progression, as evidenced by changes in synovial fluid (SF)-exosome-lncRNA Prostate Cancer Gene Expression Marker 1 (PCGEM1) levels or differences in SF-exosome-miRNAs between male and female patients [74]. miRNA, lncRNA, and circRNA are associated with the progression of OA and can serve as potential biological targets for its prevention, diagnosis, and treatment. Indeed, they play essential roles in transcriptional, post-transcriptional, and post-translational modification [75]. A complex net of exosomes is active in the joint, delivering bioactive molecules that enhance chondrocyte proliferation and ECM synthesis, inhibit synovitis, and mediate subchondral bone remodeling [76]. This regenerative capacity helps prevent further cartilage degradation, directly addressing the degenerative aspect of OA [77]. EVs from Adipose-Derived Stromal Cells (ADSCs) counteracted Interleukin-1 (IL-1β)-induced inflammatory effects on OA chondrocytes and synoviocytes by attenuating the NF-κB pathway [52].

When EVs are combined with hydrogels (Figure 2), their stability, retention, controlled release, delivery amount, regenerative potential, and therapeutic outcomes are increased [78].

Zhang et al. combined alginate–dopamine and chondroitin sulfate with a silk fibroin hydrogel sponge (AD/CS/RSF). They found that the AD/CS/RSF hydrogel with encapsulated MSC-exosomes promoted bone marrow stromal cell migration, proliferation, and differentiation, regenerated the defective cartilage, and remodeled the ECM in an animal model [79].

Wu et al. developed an injectable thermosensitive hydrogel by adding β-glycerophosphate to chitosan, a natural polysaccharide [80]. Thermosensitive hydrogels have good liquidity, are easy to use, and have desirable drug-delivery properties. Then, they encapsulated the hydrogel with bone-MSC-sEVs in rat calvarial defects. The results showed that sEV-loaded hydrogels were biocompatible, exhibiting excellent thermosensitive properties and enhanced bone regeneration. In particular, the authors discovered that bone-MSC-sEVs carried microRNA-21 (miR-21) [81] that targeted Sprouty 2 (Spry2), thereby promoting angiogenesis. The primary function of microRNAs is to downregulate the translation of target genes by preventing the specific translation of mRNA through ribosomes, thereby inhibiting protein synthesis. Indeed, miR-21 inhibited Sprouty2 (Spry2), known to downregulate the angiogenesis process [80].

Cheng et al. found that Nidogen 1-enriched EVs loaded into a composite hydrogel accelerated angiogenesis and bone regeneration in an in vivo femoral defect model. Nidogen 1 is an ECM component of the vascular basement membrane that supports vascular and endothelial integrity [82].

Engineering Exos carrying Kartogenin (KGN) have displayed potential for restoring cartilage. KGN induces the differentiation of MSCs into chondrocytes, stimulates cartilage nodule formation, and enhances cartilage regeneration. KGN’s mechanisms of action involve regulating key transcriptional programs, such as the CBFβ-RUNX1 pathway, and influencing the expression of genes involved in cartilage formation, like Acan, Sox9, and Col2a1. Shortcomings include the inhomogeneous distribution of Exos at the injury site and the release of cargo out of chondrocytes. The combination of immune regulatory Exos with 3D bioprinting hydrogel (3D-BPH-Exos) holds the potential for restoring and immunomodulating cartilage tissue and reducing inflammation of OA. In particular, there can be reduced intracellular inflammasome formation and released inflammatory agents such as IL-1β, TNF-α, and IFN-γ, while also inhibited chondrocyte apoptosis by restoring mitochondrial function and enhancing chondrogenesis in synovial MSCs, osteoprogenitor cells, and osteoclasts [83].

### 4.5. Growth Factors Combined with Hydrogels for OA Treatment

Whether natural or synthetic, GFs play a crucial role in development, tissue maintenance, and healing; they are essential to regenerative processes in joint tissues [84]. Natural GFs typically have short half-lives and low stability under physiological conditions. Only a few synthetic GFs are commercially available for clinical use, like recombinant human BMP-2 (rhBMP-2), which promotes bone formation and has received FDA approval for orthopedic treatments [85].

The biological effects of GFs depend on several aspects, including the concentration of the molecules, their binding forms, spatial distribution, and the types of receptors and cells involved. Improper administration, dosage, or release of GFs can result in unwanted tissue formation, tissue inflammatory reactions, and tumor development. For instance, high doses of bone morphogenetic protein-2 (BMP-2) have been associated with ectopic bone tissue formation [86].

Hydrogels can prevent the rapid enzyme proteolysis or deactivation of GFs and decelerate their release. GF-loaded hydrogels have shown great potential for bone regeneration [87]. However, their low mechanical strength severely limits their applications in bone regeneration [84].

Several studies investigated hydrogels delivering Bone Morphogenetic Proteins (BMPs) [87,88], enclosed in the transforming growth factor-beta (TGF-β) superfamily [89], which regulate many developmental and physiological processes. For example, BMP-6 and BMP-7-loaded hydrogels have been reported to be effective for bone regeneration [84,87]. Other studies have incorporated into hydrogels angiogenic factors (vascular endothelial growth factor (VEGF) and platelet-derived growth factor (PDGF)), together with BMP-2, to orchestrate angiogenesis [84], which is compelling for the osteogenic process [87,90].

Various hydrogels delivering TGF-β1, TGF-β3, and insulin-like growth factor (IGF-1) for cartilage regeneration have been explored [84,87].

#### Platelet-Rich Plasma

Platelets are crucial homeostasis regulators, promoting angiogenesis, modulating inflammation, and facilitating tissue regeneration. When isolated from peripheral blood, they are an autologous source of more than 1500 bioactive factors, including GFs, immune system messengers, and enzymes [91], which are crucial for tissue regeneration. The concentrated platelets can provide six to eight times the standard physiological doses of GFs, the key mechanism that stimulates healing. Additionally, the GFs released from platelets promote the recruitment and differentiation of MSCs and other target cells involved in the healing process. Since earlier studies showed that these GFs support tissue regeneration, platelet concentrates have been utilized in regenerative medicine [92].

PRP is obtained through autologous blood centrifugation, which separates erythrocytes from platelets. Before administration, PRP is combined with an activator that prompts the platelets to release various GFs from their granules [92]. These GFs, including TGF-β, VEGF, fibroblastic growth factor (FGF), and PDGF, support cell proliferation and promote osteogenic and chondrogenic differentiation. Due to these beneficial properties and the essential role of platelets in OA, there is increasing interest in using PRP as a therapeutic strategy for this condition. While PRP is clinically used for OA treatment, its efficacy remains a topic of debate. Notably, exosomes derived from PRP have shown the potential to be more effective than PRP alone in protecting cartilage. Zhao et al. investigated the therapeutic effect of intra-articular injection of PRP and PRP-derived exosomes on cartilage defects in rat knee joints. The results showed that PRP-exosomes and PRP could promote cartilage defect regeneration and inhibit tissue degradation, but the effects of PRP-exos were significantly better than PRP [93].

Various biomaterials, including hydrogels, have been studied for their ability to promote the sustained release of GFs from PRP or Platelet-Rich Fibrin (PRF) (whose preparation protocol does not require any anticoagulants) [94], thereby aiding bone and cartilage regeneration [84] (Figure 2). Evidence, for instance, shows that silk fibroin (SF) hydrogels are candidates for use in controlled drug delivery systems thanks to their high encapsulation rate and stable network structure. Jiraboonsri et al. explored the application of PRP derived from patients suffering from OA in combination with SF hydrogel for the first time. The approach involved injecting the SF-PRP hydrogel directly into the affected joints of patients with OA. The results demonstrated no evidence of toxicity associated with the treatment, and further analysis indicated that the hydrogel not only facilitated the release of GFs from PRP but also promoted chondrocytes’ proliferation and activity [95].

## 5. 3D/4D Bioprinting

Hydrogels share features such as natural extracellular membrane components and allow for cell encapsulation; therefore, they are suitable for 3D bioprinting applications. Three-dimensional bioprinting creates organized, living constructs via a “layer-by-layer” deposition of small units of biomaterials and cells. These small units are referred to as “bioinks” or “cell inks” as they incorporate cells with some substrate that the cells are either mixed with or printed onto during printing. The “bioinks “ act as a medium in which cells are suspended. They also allow for printing and solidification post-print to maintain shape fidelity and mechanical support as found in the native tissue they are replacing [96].

There are three primary categories of bioprinting techniques: inkjet, extrusion, and laser assisted. The two most used hydrogel-based bioprinting modalities are jet-based and extrusion-based printing.

Extrusion bioprinting utilizes pneumatic pressure or a syringe pump to continuously push material through a micro-nozzle [96].

A significant advantage of using extrusion-based bioprinting is the ability to select a greater range of biomaterials with a significantly wider viscosity than other bioprinting techniques. However, the forceful extrusion of high-viscosity cell inks can cause cell membrane damage. Such a consequence must be counteracted by increasing the nozzle diameter, thereby increasing cell viability [96].

Several hydrogels are compatible with extrusion-based bioprinting. Due to their liquid components, the fabrication of a 3D construct may lack biomechanical properties. The addition of crosslinking methods can improve the strength of the construct [96].

Jet-based bioprinting, also known as drop-on-demand (DOD) printing technology, deposits small droplets of cell ink, outputting a volume of between 1 and 100 picoliters with a droplet diameter in the range of 10–50 μm, in a predefined geometry on a substrate or dish. Thermal and piezoelectric approaches are commonly used. Piezoelectrically driven inkjet bioprinters utilize piezoelectric crystals in the printer tip, generating acoustic waves that propel the cell ink through the nozzle. An inkjet bio-printer driven by a thermal process heats a small volume of the cell ink to the temperature range of 200–300 °C for a few microseconds to form a small bubble of vapor. The resulting pressure forces a small volume of the cell ink through the nozzle, depositing a controlled spray of cell ink directly onto a dish or into a substrate, such as a hydrogel. The aforementioned high temperatures raise concerns about protein denaturation and cell stress, which in turn affect cell viability. Despite these concerns, several sources present results indicating high cell viability post-print, suggesting that cells experience less stress than previously presumed due to the short period of exposure to high temperatures (approximately 2 μs). However, this may depend on the cell type. The most central problem in jet-based bioprinting is that the cell ink must be able to adopt a range of states: during printing, it must be liquid to enable subsequent jetting, and post-print, it must solidify into a 3D structure that maintains the desired form and provides a habitable environment for cells. Additionally, cell inks are often limited by their viscosity; cell inks with dynamic viscosities lower than 10 mPas have been reported to be compatible with inkjet printing, a smaller range than what is possible with extrusion-based bioprinting. In comparison with other methods, inkjet printing has the downside of low cell densities. Cell concentrations are limited due to the small orifice at the tip of the printer head. The tips of these printers tend to clog, thus limiting the types of hydrogels and cell viscosities that can be used with this printing modality. Low-viscosity inks are desirable for jet-based printers but have the troublesome side effect of cell sedimentation. In addition, though the motion of the printhead itself is swift, the volume extruded is lower than what is possible using an extrusion-based bioprinter. Thus, it still can take a substantial amount of time to deposit enough cell ink to generate a structure of usable size. However, jet-based bioprinting offers numerous benefits, including the use of multiple potential cell inks and non-contact printing, which limits substrate contamination, provides control and precision, and offers considerable flexibility regarding the printed geometry [96].

3D bioprinting has opened new perspectives in musculoskeletal tissue regeneration, allowing for more precise mimics of the tissue’s real structure than conventional manufacturing methods and finer modulation of mechanical and chemical properties. Three-dimensional bioprinting enables the creation of customized construct hydrogels tailored to individual patients and heterogeneous, complex anatomical structures.

A considerable investment in assets has been dedicated to the advancement of hydrogels suitable for local injections, designed to foster the restoration of soft tissues, such as cartilage, which have notable healing challenges. These materials can be fine-tuned to mimic the physical and chemical characteristics of the repaired tissues. Doing so generates surroundings that closely resemble the body’s natural extracellular matrix. This is beneficial to the proliferation and differentiation of the encapsulated cells.

**Figure 5:**
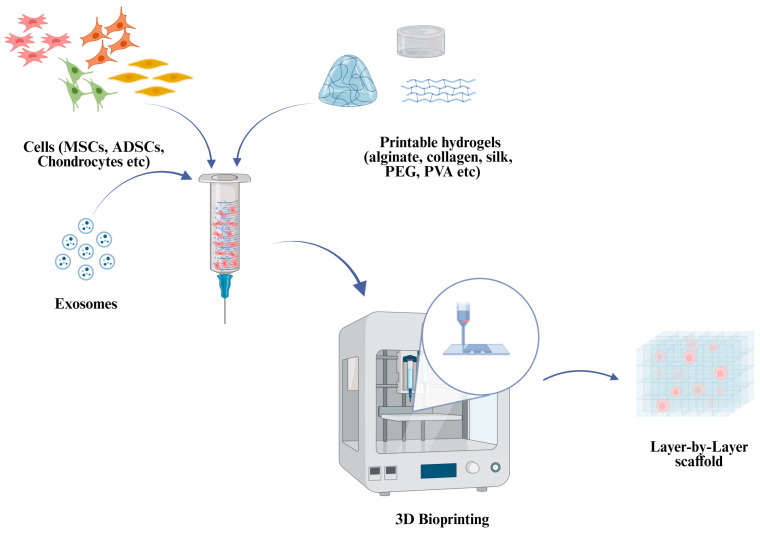
Three-dimensional Bioprinting of different orthobiologics with suitable hydrogels for future tissue engineering applications. The image was created with BioRen-der.com (Website homepage: https://www.biorender.com/, accessed on: 12 May 2025).

To date, hydrogel materials used as bioink involve natural materials, including alginate, hyaluronic acid collagen, and silk fibroin. These compounds could be combined with the different orthobiologics reported above to be used in joint regenerative treatments [97]. (Figure 5).

Recently, 4D bioprinting applications, including shape-morphing hydrogels, exhibit great potential to develop flexible scaffolds and provide a more favorable environment for healing and tissue regeneration [98]. These scaffolds may undergo form changes as the tissue regenerates. Four-dimensional bioprinting has the potential to completely revolutionize regenerative medicine by producing dynamic, responsive tissues that closely resemble the behavior of biological systems. However, many significant obstacles must be solved, like biocompatibility concerns, immunological reactions to smart materials, and undesired bodily deterioration [99].

A summary of the results from using different combinations of orthobiological and hydrogels for OA treatment is presented in Table 2. Table 3 shows the pros and cons of combining orthobiologics and hydrogels.

## 6. Conclusions and Future Perspective

Utilizing orthobiologics for treating different musculoskeletal pathologies has grown significantly due to their efficacy, versatility, and minimal incidence of serious adverse effects [14,112]. The combination of these biological compounds with different kinds of biomaterials has provided a structural framework for the regeneration of various tissues, such as damaged cartilage or bone, or in the presence of pathological conditions, such as OA.

Numerous studies have showcased the promising capabilities of hydrogels in supporting orthobiologics as a treatment for OA disease, thanks to their distinctive physical, chemical, and biological properties [45]. Hydrogels that possess superlubricating properties, designed to minimize the friction experienced within joint tissues, have shown considerable promise in mitigating the discomfort associated with OA, specifically pain.

Moreover, orthobiologics combined with hydrogels provide a regenerative potential derived from their different components, facilitating the transition from heightened inflammation to restoring tissue structure and equilibrium. Hydrogels provide a suitable framework for promoting cell growth, adhesion, and proliferation. They mimic the native ECM, creating a 3D setting to direct cellular activities and drive tissue regeneration. In recent years, there has been interest in injectable hydrogels. These scaffolds have a fluid consistency before use, making them suitable for syringe administration and accommodating wounds with unusual geometries. The injected material transforms into a gel within the body, enhancing the stability of therapeutic molecules while minimizing side effects associated with systemic administration. This in situ gelling process is the basis for creating tissue scaffolds through bioengineering, facilitating cell multiplication, specialization, and attachment, and promoting new tissue development [78].

However, a proper modulation of the mechanical properties, biocompatibility, degradation rates, and potential host responses are still inadequate. Additionally, the injection process itself can impact the hydrogel’s structure and properties.

Tackling these challenges necessitates more investigation and progress in finding innovative materials and crosslinking methods that create hydrogels that possess superior mechanical traits, high biocompatibility, and degrade in a predictable time [113].

Some hydrogels are the subject of clinical investigations, intending to assess their suitability for a broader scope of medical applications [114]. Novel protein hydrogels are providing novel insights into treating cartilage inflammation [115].

Since their inception in biomedical science, hydrogels have evolved towards breakthroughs in drug delivery. Current research focuses on modifying their characteristics and developing new ones for targeted use. Considering the current progress and developments, new characterization and modeling techniques will be necessary to support the systematic exploration of hydrogel applications. Future studies on hydrogels have the potential to significantly improve the area of drug delivery. There is a trend towards tailored therapies, as in the case of OA, to address individual lesion characteristics and disease severity. However, there are still issues to fix, such as the design of the proper mechanical and rheological properties.

Bioprinting offers a powerful tool for creating personalized therapies based on orthobiologics combined with hydrogels, potentially revolutionizing the way we manage musculoskeletal diseases and ultimately leading to improved patient outcomes.

The next decade will see the application of tissue engineering research findings, which will require excellent study and collaboration between researchers and physicians.

## Figures and Tables

**Table 2:** Orthobiologic-laden hydrogels for joint tissue regeneration.

Orthobiologic	Hydrogel	Preparation Technique	Results	Ref.
Chondrocytes	Chitosan hydrogel–demineralized bone matrix.	Chitosan powder is dissolved in 0.1 M acetic acid to yield 2.5 2.5% (*w*/*v*) aqueous solution and then sterilized in an autoclave. The CS solution is mixed with filter-sterilized and pre-cooled 60% (*w*/*v*) GP at a ratio of 9:1 to obtain homogeneous CS/GP solutions	The study demonstrated that the transplantation of allogenic chondrocytes with chitosan/DBM scaffold successfully repaired rabbit cartilage injury with only one-step operation	[52]
Chondrocytes	α-cyclodextrin and polyethylene glycol (PEG) incorporated into a gelatin covalent matrix	The preparation of PEG/α-CD polyrotaxanes involves dissolving two different concentrations of α-cyclodextrin (α-CD) (12 and 36 mg) in 400 μL of PBS. This solution is then added to a 600 μL PEG/PBS solution (6.5 wt%)	By incorporating supramolecular motifs into a covalent-based hydrogel system, it is possible to mimic the intricate dynamic and noncovalent interactions present in natural cartilage	[53]
Chondrocytes	Photo-crosslinking, zwitterionic	Three different monomer mixtures were prepared and are denoted as follows: MPC60, MPC30, and SBMA60. The monomer solutions were polymerized into hydrogels for 10 min using a Bluephase G4 polymerization lamp	The results demonstrated that zwitterionic cartilage–hydrogel networks are formed by infiltration The applied hydrogels could completely infiltrate the human cartilage explants and still be polymerized by visible light	[54]
MSCs (from bone marrow, adipose tissues, or other sources)	HA, agarose, alginate, glycosaminoglycans, and chitosan, proteins like collagen, elastin, gelatin, polyvinyl alcohol (PVA), or Poly(lactide-co-glycolide) (PLGA)	Conventional subtractive or innovative additive manufacturing (3D printing)	Choncrogenic/osteogenic effects	[57,58]
SVF	HA/gelatin-biphasic calcium phosphate (HA-Gel/BCP)	The BCP scaffold was prepared by the sponge replicate method. The gelatin was dissolved and mixed with HA. Then, the Ha-Gel solution was added to the BCP. Finally, the HA-Gel-loaded BCP scaffold was crosslinked using 1-ethyl-3(3-dimethylaminopropyl)carbodiimide hydrochloride and N-hydroxyl succinimide	The isolated SVF showed osteogenic differentiation ability. In vivo implantation of autologous SVF-hydrogel complex showed excellent bone regeneration in a rat skull critical-size defect model	[61]
Exosomes (MSC-derived)	Alginate–dopamine and chondroitin sulphate with a silk fibroin hydrogel sponge (AD/CS/RSF)	Hydrogel preparation encompasses using a crosslinked network	The hydrogel with encapsulated exosomes recruited BMSCs and promoted cartilage regeneration	The AD/CS/RSF/EXO hydrogel
Exosomes containing miR-21targeted SPRY2 (Bone-MSC-derived)	Thermosensitive β-glycerophosphate/chitosan	Chitosan was added to an acetic acid solution, followed by the dropwise addition of β- glycerophosphate solution	The exosome–hydrogel complexes were biocompatible, exhibiting excellent thermosensitive properties, and enhancing bone regeneration	[80]
Nidogen1-enriched Exosomes (Bone marrow-MSC-derived)	Alginate/PEG2000/gelatin composite	A mixed prepolymer solution (Sodium alginate, PEG2000, and gelatin) was added with exosomes carrying Nidogen-1 and transferred to a mold and solidified. Then, 2% CaCl_2_ solution was used to crosslink	Nidogen1-enriched Exosomes derived from BMSCs promote angiogenesis and bone regeneration in rat femoral defects	[82]
Krthogenin-enriched Exosomes (Umbilical cord-MSC-derived)	In situ HA hydrogels achieving gelation by imine-mediated ortho-nitrobenzyl alcohol- photo crosslinking	Bioprinting	Krthogenin-enriched exosomes reduced intracellular inflammasome formation and the release of inflammatory agents, while preventing chondrocyte apoptosis and enhancing chondrogenesis in synovial MSCs, osteoprogenitor cells, and osteoclasts in OA	[83]
Growth factors (BMP-2, BMP-6, BMP-7, TGF-β1, TGF-β3, IGF-1, VEGF, PDGF)	Natural, synthetic, and composites	Recombinant process	Increase cell proliferation and differentiation; promote ECM synthesis	[87]
PRP	Silk fibroin	A fibroin solution of 4% aseptically prepared medical grade Thai silk was sonicated and solutions were mixed with PRP at the ratio of 1:1, giving the final con-centrations of SF. The mixtures were then left to gelate	Silk Fibroin-PRP complex can be easily injected, releasing GFs in a sustained manner. It is biocompatible, able to promote chondrocyte proliferation, and is not cytotoxic	[95]

**Table 3:** Pros and cons of orthobiologic–hydrogel combination.

Pros	Cons
**Enhanced Tissue Regeneration** Hydrogels provide a 3D scaffold for orthobiologics, mimicking matrix structure, that supports cell survival, differentiation, and integration into the damaged tissue, and potentially creates the condition for the regeneration of damaged joint tissues regeneration [100].	**Degradation and Integration** Hydrogels can precociously or partially integrate with the surrounding joint tissue, affecting long-term effectiveness [101].
**Controlled Delivery** Hydrogels’ design can be tailored to control orthobiologics release, and therefore their therapeutic effect [102].	**Cell Limitations** Chondrocytes can be difficult to culture and expand in sufficient numbers, and they may not always differentiate into the desired hyaline cartilage [103]. **MSCs can undergo hypertrophic differentiation** During the physiological process of cartilage formation, MSCs differentiate into chondrocytes, which then differentiate into hypertrophic chondrocytes that produce Type X collagen and mineralize. In the context of cartilage regeneration using MSCs, an undesired hypertrophic differentiation can happen, leading to bone, but not cartilage formation.
**Minimally Invasiveness** Injectable hydrogels offer a less invasive approach compared to some surgical procedures for joint tissue repair [104].	**Immunogenicity** Some hydrogel materials may trigger an immune response, leading to inflammation and rejection [105].
**Improved Adhesion** Some hydrogels can adhere to joint tissues, improving their retention at the treatment site [106].	**Cost and Complexity** Developing and manufacturing hydrogels and cell-based therapies can be expensive and complex [13].
**Reduced Inflammation** MSCs, concentrates, exosomes, and growth factors possess immunomodulatory properties and reduce inflammation in the joint, a key factor in OA progression [107].	**Limited Long-term Results** Clinical studies on safety and efficacy orthobiologic–hydrogel combinations mostly concern short term evaluations. Long-term clinical results are still needed to support the use of those strategies [108].
**Targeted Delivery** Hydrogels can be engineered to deliver Orthobiologics to the site of joint tissue damage, maximizing their therapeutic effect and minimizing potential side effects due to systemic administration [13,100].	**Cell Viability and Survival** Cells can suffer from the hostile, inflamed OA joint, thus hampering a proper engraftment in the site of damage [13].
**Improved Mechanical Support** Hydrogels must mimic the mechanical properties of joint tissue to improve weight bearing and mobility [109].	**Hydrogel Degradation** Hydrogels can degrade over time, potentially leading to the release of cells before they have fully integrated into the tissue [109].
**Potential for Long-Term Results** While more research is needed, some studies indicate that orthobiologics therapy, particularly when combined with hydrogels, may offer long-term benefits for OA patients by promoting sustained joint tissue repair and reducing disease progression [47].	**Mechanical Properties** The mechanical properties of hydrogels must ensure the engraftment to support the stresses and strains of the joint [109].
**Improved Pain Relief** The combination of orthobiologics with hydrogels can reduce pain associated with OA, likely due to their ability to modulate the inflammatory environment and promote tissue regeneration [110].	**Need for Standardization** Standardized protocols for orthobiologic preparation and delivery are still scarce, which can affect the consistency and reproducibility of the results [111].

## Data Availability

No new data was created.
